# Narrative review of celiac disease and sports: nutritional deficiency and strategies to optimise athlete health and performance

**DOI:** 10.1016/j.crfs.2026.101337

**Published:** 2026-02-01

**Authors:** Alessia Boatta, Giuseppe Messina, Andrea Pagliaro, Anna Alioto, Omar Mingrino, Domenico Nuzzo, Pasquale Picone, Patrizia Proia

**Affiliations:** a Department of Human Sciences and Promotion of the Quality of Life, San Raffaele University, Rome, 00166, Italy; b Institute for Biomedical Research and Innovation, CNR, via U. La Malfa 153, Palermo, 90146, Italy; c Department of Psychology, Educational Science and Human Movement, Sport and Exercise Sciences Research Unit, University of Palermo, Palermo, 90128, Italy

**Keywords:** Celiac disease, Sport, Athletic performance, Gut microbiota, Sports nutrition, Nutritional deficiencies

## Abstract

Celiac disease is an autoimmune disorder that compromises the integrity of intestinal mucosa and impaired nutrient absorption. Gluten-free diet is currently the only available therapy, but it is not always sufficient to ensure adequate nutritional intake and may be associated with micro- and macronutrient deficiencies, with potential metabolic and functional consequences, particularly in athletes with high energy demand. Indeed, athletes with celiac disease are at increased risk of developing nutritional imbalances, which may have a harmful effect on both intestinal health and performance. In recent years, the inclusion of naturally gluten-free cereals and pseudocereals, in conjunction with bioactive compounds such as polyphenols, has been demonstrated to enhance the nutritional quality of a gluten-free diet. In addition, probiotics supplementation shows promise in improving gut health, modulating inflammation, contributing to better recovery and performance. However, gaps in evidence persist, particularly concerning high-performance athletes. Therefore, this review integrated clinical and nutritional evidence, including a few available trials on athletes with celiac disease to provide an updated synthesis of how gluten-free diets, microbiota modulation, and nutritional strategies influence performance, recovery, and metabolic health, highlighting future directions for evidence-based interventions in sport to optimise athlete health and performance.

## Highlights

• Celiac athletes are at risk of nutrient imbalances that can affect performance.
• Gluten-free cereals and polyphenols can improve athletes ‘diet quality.
• Probiotics support gut health, reduce inflammation, and aid recovery.
• A personalised, multidisciplinary line is important for managing celiac athletes.

## Graphical abstract

**Figure:**
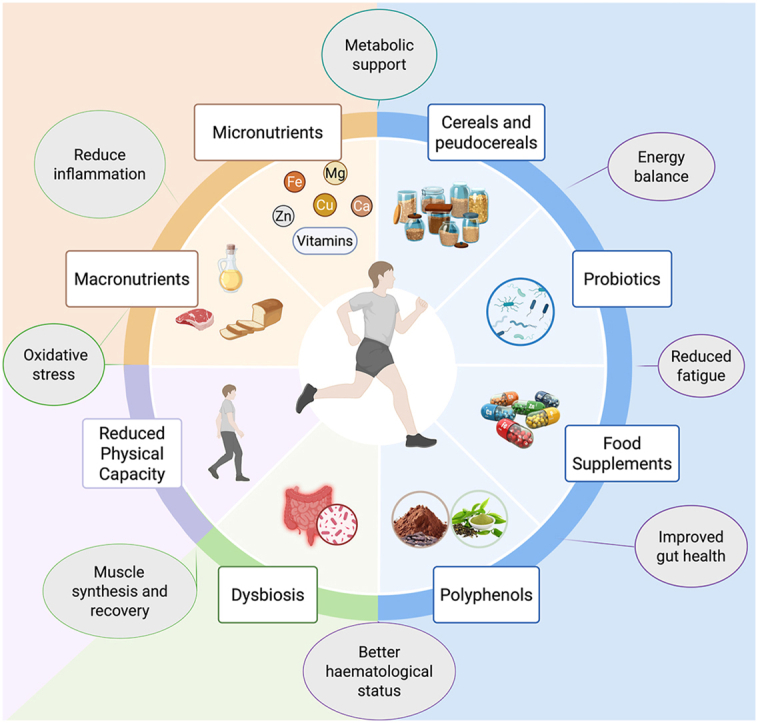

## 1 Introduction

Celiac disease is a chronic autoimmune disorder triggered by gluten ingestion in genetically predisposed individuals that primarily affects the small intestine, leading to nutrient malabsorption (Ben Houmich and Admou, 2021). Compared with the general population, athletes have higher metabolic and nutritional requirements and, in the presence of celiac disease, these demands may be difficult to meet due to impaired nutrient absorption and the restrictive nature of a gluten-free diet, which can hinder their ability to sustain intense physical activity (Leone et al., 2020). Athletes, generally, require optimised energy and nutrient intake to sustain training and enhance recovery. Indeed, micronutrients such as iron, calcium, magnesium, zinc, D and B vitamins and macronutrients as carbohydrates and proteins plays an important role in energy metabolism, muscle repair, and immune regulation. In athletes with celiac disease, intestinal villous atrophy leads to malabsorption of these nutrients, which may cause fatigue, anaemia, reduced energy availability, muscle weakness and impaired recovery (Damian et al., 2021). These consequences are important for endurance and strength athletes, where oxygen transport, mitochondrial function, and protein synthesis are performance determinants. The only effective therapy for celiac disease is lifelong adherence to a gluten-free diet. However, if it is not planned carefully, it can potentially cause nutritional deficiencies and alter the gut microbiome. Indeed, gluten-free products are often characterised by unbalanced nutrient composition, with higher glycaemic index with increased saturated fat and carbohydrate content, lower protein and fibre content, and reduced levels of key micronutrients compared to gluten-containing equivalents (Cadenhead et al., 2024). These factors can all negatively affect recovery, muscle growth, energy availability and can compromise glycogen replenishment, muscle synthesis, and energy metabolism, reducing endurance and performance potential (Myhrstad et al., 2021). In this sense, athletes with celiac disease have to deal with two challenges: maintaining intestinal integrity while meeting the nutritional demands required for optimal athletic performance. Several studies have reported that undiagnosed or poorly managed celiac disease can contribute to decreased physical capacity, recurrent fatigue, and a higher risk of injury among athletes (Therrien et al., 2020). Iron deficiency and anaemia are among the most common complications and are strongly associated with chronic fatigue and decreased oxygen transport to working muscles (Moum and Lindgren, 2025). This is particularly important for endurance athletes, where aerobic efficiency and mitochondrial function are key determinants of performance. In addition, persistent inflammation and increased intestinal permeability in celiac disease can impair recovery and exercise-induced oxidative stress, further compromising training adaptation (Sim et al., 2019; Stefanelli et al., 2020). Recent studies explored strategies to improve the nutritional quality of gluten-free diets for athletes (Arslan et al., 2019). Additionally, emerging evidence highlights the importance of gut microbiota in modulating nutrient absorption, inflammation and exercise metabolism. In this context, supplementation with probiotics has attracted attention as a potential tool for celiac athletes. Indeed, restoring microbial balance, improving intestinal barrier function, and reducing systemic inflammation may enhance recovery and performance (Pecora et al., 2020; Teglas and Radak, 2025). At the same time, supplementation with polyphenols, bioactive compounds present in plant-based foods, acts with their antioxidant and anti-inflammatory effects that could mitigate exercise-induced oxidative damage and promote intestinal health. In addition to dietary modifications, individualised supplementation protocols may be required to meet the increased nutritional demands of training while correcting deficiencies common in celiac disease (Cannataro et al., 2023). Despite an increase in public interest in gluten-free diets and sports, scientific evidence on the prevalence and performance outcome of celiac disease in athletes remains poorly explored. In a cross-sectional study of collegiate athletes in the United States, Leone et al. (2020) found that athletes were 3.85 times (95% CI, 0.42–34.89) more likely to report a celiac disease diagnosis and 18.36 times (95% CI, 2.40–140.48) more likely to report high levels of celiac disease symptoms than the general population. However, the study also revealed a lack of awareness, diagnosis and management of the condition among athletes. Furthermore, athletes with higher Celiac Symptom Index (CSI) scores reported a significantly lower quality of life, as well as higher levels of perceived stress and depressive symptoms. These results imply that celiac disease and related gastrointestinal disorders may be underdiagnosed or underestimated in athletic populations, which could affect psychological well-being and athletic performance. More recently, Cannataro et al. (2023) reported the first case of structured nutritional management of a high-level swimmer with celiac disease, showing that a targeted nutritional intervention aimed at optimising energy and protein intake, macronutrient quality and timing of intake in relation to training, combined with specific supplementation, can lead to improvements in body composition, metabolic parameters and athletic performance. One of the major findings of the author was that a gluten-free diet alone may not be sufficient for high-level athletes with celiac disease and this highlights the importance of personalised dietary and supplementation strategies in restoring energy balance and physical performance. Beyond these two studies, no clinical trial or intervention data are currently available in the literature. This review is therefore the first to comprehensively integrate current nutritional evidence on celiac disease and athletic performance. Specifically, it aims to summarise the physiological and metabolic consequences of celiac disease in athletes, evaluate the impact of gluten-free diet on performance, recovery and gut microbiota, analyse available clinical data and case reports and propose emerging strategies to support health, recovery and quality of life in celiac athletes.

### 1.1 Impact of celiac disease on athletic performance

The HLA-DQ genotype is a determining factor in the development of celiac disease and can also impact the composition of gut microbiota, which in turn affects nutrient absorption, immune response, and intestinal integrity (Bascuñán et al., 2020; Pecora et al., 2020). Gut microbiota is important not only for gut health, but also for supporting physical performance by helping to maintain an optimal balance for nutrient absorption and immune system function. Studies have shown that athletes and physically active people have a greater microbial diversity in the gut, with a higher prevalence of beneficial species, and increased microbial metabolic activity, particularly in pathways related to carbohydrate and amino acid metabolism, compared to sedentary individuals (O'Brien et al., 2022). Regular endurance activity positively modulates the composition of gut microbiota, reducing the presence of pro-inflammatory bacteria such as *Proteobacteria*. Beyond physical activity, the composition and quality of macronutrients, carbohydrates, proteins and fats have a significant impact on the gut microbiome. To optimise sports performance, athletes must consume an adequate amount of simple carbohydrates, such as glucose, fructose and sucrose, before and during exercise, to maintain glucose homeostasis, reduce fatigue, facilitate rehydration and ensure optimal water balance (Chen et al., 2024). Fibre, in particular, promotes a stable microbial composition, enhancing intestinal immunity and reducing the risk of dysbiosis. Insufficient fibre intake, on the other hand, can compromise gut health and the efficiency of microbial metabolism (Chen et al., 2024). However, a gluten-free diet may negatively affect microbial composition by reducing the presence of beneficial bacteria in favour of potentially harmful strains, promoting dysbiosis (Akobeng et al., 2020; Serena et al., 2020; Aboulaghras et al., 2022). In this context, nutritional interventions and probiotic supplementation can support gut microbiota and sport performance in athletes with celiac disease. Probiotics can restore microbial balance, improve nutrient absorption, and reduce inflammation and physical fatigue, as well as mitigate the inflammatory response following intense exercise. Indeed, modulating intestinal microbiota with probiotics can mitigate muscle damage (Sausa et al., 2024). Combining probiotics with macronutrient intake can support microbiota activity, energy availability and recovery.

In celiac disease, intense physical activity can compromise nutrient absorption and impair performance. Endurance training increases metabolic demands and, in the presence of celiac disease, the absorption of iron, protein and other micronutrients may be compromised, which can lead to fatigue and reduced physical capacity (Damian et al., 2021). Gastrointestinal alterations typical of celiac disease can increase iron loss in the digestive tract, damage the intestinal mucosa, increase permeability and slow down gastric and intestinal motility, thereby increasing the risk of anaemia in athletes (Damian et al., 2021). Therefore, athletes with celiac disease are particularly susceptible to exercise-induced fatigue and suboptimal recovery (Therrien et al., 2020; Skjellerudsveen et al., 2023; Bouery et al., 2024). A gluten-free diet and a targeted nutritional intervention can restore adaptive and innate immune parameters, supporting nutrient absorption to meet the increased training demands(Usai Satta et al., 2024).

## 2 Celiac athletes diet

For athletes with celiac disease, adhering to a strict gluten-free diet is essential for intestinal healing and optimal nutrient absorption. However, gluten-free products typically contain rice or maize flour and commercial starches instead of wheat flour, which often results in lower nutritional quality and reduced acceptability. These products tend to contain lower levels of protein, dietary fibre, fat, sodium, and micronutrients such as iron and folate, while containing higher levels of rapidly digestible carbohydrates than their gluten-containing counterparts. Combined with this nutrient profile, the lower dietary fibre intake frequently observed in celiac athletes may compromise energy availability, recovery and muscle synthesis during training. Dietary fibre, also known as roughage, is the edible part of plant foods that cannot be digested in the small intestine. It is mainly composed of polysaccharides, oligosaccharides and associated plant compounds that provide numerous health benefits. Adequate hypertension, stroke and certain gastrointestinal disorders. Higher fibre consumption also support immune function, helps maintain healthy serum lipid levels, promotes weight management, lowers blood pressure, improves glycemic control and supports regular bowel function. The recommended fiber intake ranges from 25 to 35 g per kilogram of body weight per day, underlining the importance of enhancing gluten-free products to increase their nutritional quality because they generally have low amounts of fiber (Arslan et al., 2019). Also, gluten-free products tend to have a higher glycaemic index, which can contribute to the development of metabolic syndrome, a group of conditions that increase the risk of cardiovascular disease (Motazedian et al., 2023; Wang et al., 2023). It is important to understand these limitations when planning dietary strategies to reduce nutritional deficiencies, maintain performance and support recovery.

Athletes have nutritional demands that are closely related to the type, intensity, and frequency of physical activity. Inadequate energy availability can impair performance and lead to adverse effects on several physiological systems, including reduction of lean mass, immune dysfunction, loss of bone mineral density, and increased risk of injury and overtraining symptoms. Adequate energy is provided by carbohydrates, which are the main substrate for endurance exercise. They are stored as glycogen in the muscles and liver, and must be replenished promptly following exercise. Carbohydrate intake ranges from 3 to 12 g/kg body weight/day depending on training intensity. It is recommended to consume 1.0–1.5 g/kg/h immediately after exercise and to continue for approximately 6 h, as delaying consumption can reduce glycogen synthesis by 45%. Protein is also essential as it stimulates muscle protein synthesis, promotes the maintenance of lean mass and aids recovery after exercise. To this end, it is essential to ensure an adequate supply of proteins of high biological value, rich in essential amino acids, such as those of animal origin. The required amount of protein varies depending on the sport and goals, ranging from 1.4 to 2.0 g/kg for endurance sports to over 3 g/kg during the definition period for strength athletes. It is recommended to assume approximately 0.31 g/kg of highly digestible protein within 2 h after training (Naselli et al., 2024). Fats are also an important source of energy, especially during long periods of physical activity. However, a high intake of lipids does not provide significant performance benefits. Among lipids, omega-3 fatty acids are of particular interest to athlete because of their ability to modulate the inflammatory response, reduce muscle soreness and limit oxidative stress. Fat should account for 20–35% of total calorie intake, with saturated fat making up less than 10% of this. Finally, vitamins and minerals, although not directly ergogenic, are essential for the proper functioning of energy metabolism, bone health, immune and reproductive functions. Finally, micronutrient requirements may be increased due to intense physical activity, sweating, urinary losses and dietary restrictions (Amawi et al., 2023; Naselli et al., 2024).

## 3 Nutritional deficiencies and effects on health

Although a gluten-free diet is essential for managing celiac disease, it has some nutritional limitations, which are particularly relevant for athletes with higher energy and nutrient demands (D'Angelo et al., 2020; Jivraj et al., 2022; Cannataro et al., 2023). Deficiencies in iron, vitamins, folic acid, zinc, copper and reduced calcium absorption are frequently observed in athletes with celiac disease (Cardo et al., 2021; Jivraj et al., 2022). Effective strategies to counteract malabsorption and deficiencies in micronutrients and vitamins may include adequate hydration and antioxidant supplementation to improve intestinal function and reduce oxidative stress (Damian et al., 2021; Świercz et al., 2023). Proteins are essential for satiety, thermogenesis and maintaining muscle mass, and a deficiency can compromise overall metabolic well-being (Abdi et al., 2023). Prioritising naturally gluten-free foods can improve the overall nutrient profile, and when combined with targeted supplementation to address specific deficiencies, it can enhance athletic performance.

**Fig. 1:**
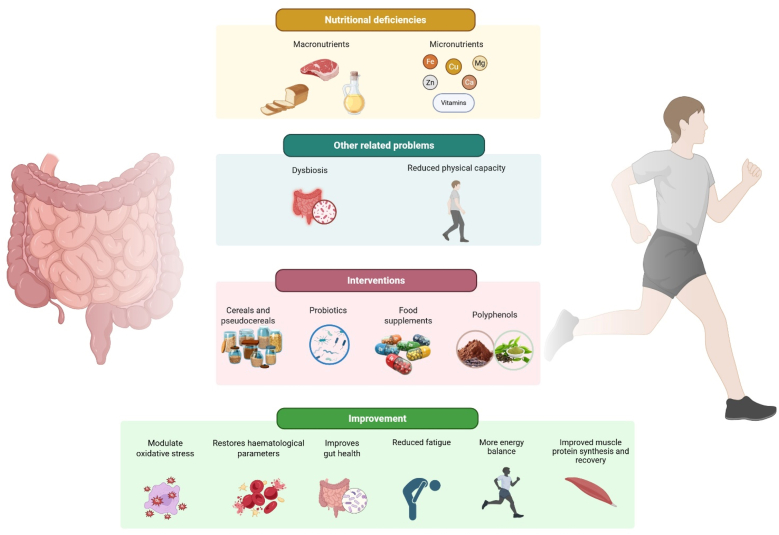
Representative image of a dietary intervention for celiac athletes. This figure provides a schematic overview of a comprehensive dietary intervention for athletes with celiac disease, integrating a balanced gluten-free diet with targeted nutritional strategies to support intestinal health, metabolic balance, and athletic performance. The framework emphasises the role of high-quality gluten-free foods, adequate macronutrient distribution, correction of micronutrient deficiencies, and the use of functional bioactive compounds such as probiotics and polyphenols. Together, these elements contribute to improved gut integrity, reduced inflammation, enhanced recovery, and optimisation of physical performance in athletes with celiac disease. Created in https://BioRender.com.

Nutritional deficiencies in celiac disease can result both from the disease itself, which damages the absorptive surface of the small intestine, and from the gluten-free diet, which can be deficient in various nutrients. Studies showed that micronutrient deficiencies persist even in celiac patients who have followed a strict gluten-free diet for more than two years. Deficiency rates include 30% for vitamin B_12_, 40% for iron, 20% for folic acid, 25% for vitamin D, 40% for zinc, and in children, 3.6% for calcium and 20% for magnesium (De la Calle et al., 2020; Alkalay, 2022). For athletes, these nutrients are essential to support physical performance and recovery. Vitamin E, through its antioxidant properties, help to reduce muscle damage and oxidative stress caused by intense physical activity, thereby improving recovery and immune function (Ghazzawi et al., 2023). Similarly, vitamin D contributes to hormone synthesis, bone health, the immune system and muscle performance. A deficiency can reduce strength, power and resistance to injury. B-complex vitamins are essential to support energy metabolism, brain function, sleep, stress management and fatigue reduction in athletes. Also, minerals support key functions as oxygen transport, heart rate regulation, antioxidant activity and bone health. Iron, essential for the production of red blood cells, is important for the transport of oxygen to muscles. Its deficiency can lead to fatigue and reduced endurance, negatively affecting physical performance. Iron also supports energy metabolism, thermoregulation and the functioning of the immune and endocrine systems. Calcium is another important mineral for athletes, particularly for muscle contraction and bone health. It helps convert carbohydrates and fats into energy. Potassium regulates water balance, blood pressure and neuromuscular function. Adequate potassium intake helps prevent cramps and reduce muscle fatigue by reducing the build-up of lactic acid. Magnesium, involved in over 300 enzymatic reactions, is essential for energy production and muscle function. Its deficiency can impair metabolic efficiency and overall athletic performance. Zinc supports endurance, strength and immune health, while also playing a fundamental role in energy metabolism and protein synthesis. It has been associated with improved oxygen utilisation and neuromuscular coordination. Finally, selenium, due to its antioxidant properties, reduces oxidative stress and exercise-induced inflammation (Ghazzawi et al., 2023). Although a gluten-free diet is fundamental for managing celiac disease, it may lead to metabolic and gastrointestinal problems. These arise from increased glycaemic index of many gluten-free products, contributing to conditions such as insulin resistance, cardiovascular disease, type 2 diabetes, constipation, diverticulitis and persistent digestive symptoms (Bjelaković et al., 2020; Cardo et al., 2021). In addition, athletes following a gluten-free diet may also be at greater risk of low energy availability, which can influence training adaptation, recovery and performance (D'Angelo et al., 2020). Prolonged low energy availability has been linked to some health problems, as reduced bone mineral density, micronutrient deficiencies, and hormonal and menstrual dysfunction (Hertig-Godeschalk et al., 2023). For these reasons, the nutritional quality of chosen gluten-free foods must be taken into account to avoid metabolic imbalances and associated health risks as type 2 diabetes, metabolic syndrome and dyslipidaemia (Marciniak et al., 2021), (Fig. 1).

## 4 Strategies to reduce the nutritional deficiencies

### 4.1 Food supplements

Cannataro et al. (2023) assessed a targeted nutritional intervention aimed at correcting deficiencies observed in celiac patients. These approaches include increasing total caloric intake and adjusting the macronutrient distribution, specifically reducing carbohydrate intake while increasing protein and fat intake. This redistribution of macronutrients was intended to support energy balance and improve nutrient absorption. In addition to dietary changes, the intervention included a structured supplementation programme designed to restore and maintain adequate levels of key nutrients for athletes, particularly vitamin B_12_, vitamin B_9_, vitamin C, and vitamin D, to address common deficiencies and support immune and gut health. Iron, magnesium, omega-3 fatty acids, creatine monohydrate and beta-alanine were also supplemented (Table 1). The results showed improvements in haematological parameters and nutrient status, with elevated levels of iron, magnesium, vitamin B_9_, vitamin D, and vitamin B_12_. There were also improvements in body weight, blood glucose regulation and systemic inflammation, indicating better metabolic balance, energy availability and recovery (Cannataro et al., 2023). This study demonstrates that targeted nutritional interventions, combining dietary planning and supplementation, can effectively improve performance, recovery and overall health of celiac athletes.

**Table 1:** Nutritional and supplementary protocol for athletes with celiac disease.

Nutrient/Supplement	Dose	Duration	Effect
Protein	2 g/kg BW	Ongoing	Support muscle synthesis and recovery
Fat	2 g/kg BW	Ongoing	Energy balance
Carbohydrates	Reduced	Ongoing	Macronutrient redistribution
Vitamin B12	1000 μg/day	3 months, then every 2 months	Correct deficiency and support metabolism
Vitamin B_9_	100 μg/day	3 months	Correct deficiency
Vitamin C	500 mg/day	Ongoing	Antioxidant and supports iron absorption
Vitamin D	3000 U/day	2 months	Gut integrity and immune modulation
Iron	30 mg/day	1 months	Correct the deficiency and improve oxygen transport
Magnesium	200 mg/day	Ongoing	Muscle function and energy metabolism
Omega-3	Softgel 60% DHA/EPA	Ongoing	Anti-inflammatory and recovery
Creatine monohydrate	3 g/day	Every 2 months	Improve strength and endurance
Beta-alanine	4 g/day	Every 2 months	Endurance performance and reduce fatigue

This table describes a comprehensive nutritional and supplementation approach developed for athletes with celiac disease, with the aim of addressing nutritional deficiencies related to intestinal malabsorption and the long-term adoption of a gluten-free diet. The protocol combines adjustments in macronutrient intake with targeted vitamin, mineral, and ergogenic supplementation to support energy availability, muscle recovery, immune function, and overall metabolic balance. Particular attention is given to meeting the elevated nutritional demands associated with intense training, while simultaneously promoting gut health and functional recovery. Adapted from Cannataro et al. (2023).

BW = body weight; DHA = docosahexaenoic acid; EPA = eicosapentaenoic acid.

### 4.2 Probiotics

The persistence of microbial dysbiosis in celiac patients, even after long-term adherence to a gluten-free diet, has promoted the exploration of additional therapeutic strategies, such as the use of probiotics. Recent studies suggest that gut microbiota and probiotics may influence the immune response in celiac disease by degrading toxic gluten peptides, modulating inflammation, and maintaining intestinal barrier integrity (Pecora et al., 2020). Probiotics are live microorganisms that, when consumed in sufficient quantities, provide a range of health benefits, especially in the context of celiac disease. Some probiotic strains can safely modulate the immune system without causing inflammation (Akobeng et al., 2020). In recent years, in fact, probiotics have been used as an adjunct to a gluten-free diet in celiac patients, with a particular focus on strains of *Bifidobacterium* and *Lactobacillus* (Jedwab et al., 2022). This choice is based on microbiota alterations, typically observed in celiac disease, including reduced beneficial bacteria, increased *Proteobacteria*, *Bacteroidetes* and *Actinobacteria*, and a *Firmicutes*/*Bacteroidetes* imbalance that a gluten-free diet may not fully correct (Marciniak et al., 2021). In particular, *Bifidobacterium breve* and *B. longum* have shown potential to reduce proinflammatory cytokines such as tumor necrosis factor-alpha (TNF-α), while promoting anti-inflammatory cytokines such as interleukin-10 (IL-10), thereby helping to limit intestinal inflammation and support microbial homeostasis (Wu et al., 2021; Jedwab et al., 2022). In vitro and animal studies have further highlighted the beneficial role of *Lactobacillus casei*, *Lactobacillus reuteri* and *Bifidobacterium longum* CECT 7347 in degrading immunotoxic gliadin peptides, suppressing inflammatory pathways such as nuclear factor kappa-light-chain enhancer of activated B cells (NF-κB) and increasing cell viability, while affecting T-cell balance and interleukin-22 (IL-22) production through activation of the aryl hydrocarbon receptor (AhR). In addition, bacterial metabolites such as butyrate, lactate and polysaccharide A from *Bacteroides fragilis* have been shown to enhance gene expression of proteins critical for gut barrier function and epigenetically influence the regulation of immune factors such as forkhead box P3 (FOXP3) (Roque and Pereira, 2024). Studies on the efficacy of probiotics have suggested that a combination of strains may be more effective at degrading gliadin peptides than a single strain (Serena et al., 2020). There is emerging clinical evidence in humans, with studies reporting that probiotic supplementation improves gastrointestinal symptoms, reduces inflammation markers, and partially restores microbial diversity. A trial involving the administration of *Bifidobacterium breve, B. animalis* subsp*. lactis, Lactobacillus casei* and *L. plantarum* for 6 weeks showed a reduction in gastrointestinal symptoms and an improvement in gut microbiota composition in adults following a gluten-free diet (Laterza et al., 2025). Interestingly, the recent evidence from Teglas & Radak (2025) further extends the role of *Bifidobacterium* and *Lactobacillus* species to sports performance, showing that probiotic supplementation can modulate lipid and energy metabolism, increase short-chain fatty acid (SCFA) production, and reduce systemic inflammation and oxidative stress in endurance and intermittent sports (Teglas and Radak, 2025) (Table 2). These findings suggest that probiotics may serve as a complementary strategy for athletes with celiac disease to enhance gut health, modulate inflammation and potentially support nutrient absorption and performance.

**Table 2:** Physiological and performance effects of probiotic supplementation in athletes.

Probiotic (strain)	Physiological effects	Type of sport	Dose
*Bifidobacterium longum* subsp. *longum* OLP-01	Increase the diversity of intestinal microbiota and improve energy substrate utilisation and lipid oxidation	Endurance	1.5 × 10^10^ CFU/day for 5 weeks
*Bifidobacterium lactis* BL-99	Enhances production of SCFA and PUFA, improves strength and lipid metabolism	Endurance	1 × 10^9^ CFU/day for 8 weeks
*Bifidobacterium animalis* subsp. *lactis* + *Lactobacillus acidophilus*	Reduces proinflammatory cytokines and increases T cells, improving immune response	Endurance	1 × 10^10^ CFU/day for 30 days
*Lactobacillus plantarum* TWK10	Increases muscle mass, reduces body fat, and improves physiological adaptation to exercise	Endurance and strength sports	3 × 10^10^ CFU/day for 6–8 weeks
Multi-strain mixture (*L. plantarum, L. casei, B. breve, B. bifidum*)	Decreases systemic inflammation and oxidative stress, improving lipid and carbohydrate metabolism	Endurance	1 × 10^9^ CFU/day for 4–12 weeks
*Bifidobacterium lactis* + *Lactobacillus acidophilus* + *B. bifidum* + *B. animalis*	Reduces fatigue and improves recovery	Strength	1 × 10^10^ CFU/day for 17 weeks

This table summarises the key physiological and performance-related outcomes associated with probiotic supplementation in athletes engaged in endurance and strength disciplines. The probiotic strains reported have been shown to positively influence gut microbiota composition, reduce inflammation and oxidative stress, and improve energy metabolism, immune function, and recovery from exercise. These findings support the emerging role of probiotics as a complementary nutritional strategy, particularly relevant for athletes with celiac disease, in whom persistent alterations of the gut microbiota may affect both nutrient absorption and training adaptation. Adapted from Teglas and Radak (2025).

CFU = colony-forming units; SCFA = short-chain fatty acids; PUFA = polyunsaturated fatty acids. Evidence from Teglas and Radak (2025).

### 4.3 Polyphenols

Recent studies suggest that polyphenols, found in many plant-based foods, may have a protective and therapeutic role in celiac disease. These bioactive compounds can bind to gliadin peptides, thereby preventing its interaction with intestinal receptors and consequently inhibiting the activation of the immune response. The effectiveness of this interaction is dependent on the polyphenol's structure and environmental pH, with catechins such as epigallocatechin gallate (EGCG) and procyanidins demonstrating greater affinity for gliadin. In addition to sequestration, polyphenols can modify gliadin's structure, thereby masking its immunostimulatory epitopes and reducing deamidation by tissue transglutaminase, thereby attenuating T-cell activation. This process has been demonstrated to assist in reducing the activation of the immune response and the maintenance of the intestinal barrier's integrity (Ribeiro et al., 2020). In vitro studies, particularly those involving green tea and cocoa polyphenols, have demonstrated their ability to reduce intestinal inflammation and improve intestinal barrier function, highlighting their therapeutic potential in the management of celiac disease (Van Buiten and Elias, 2021). Indeed, from a nutritional point of view, a daily intake of 300–500 mg of catechins or 500–700 mg of cocoa flavanols has been associated with improved antioxidant status, reduced lipid peroxidation and enhanced endothelial function. These improvements may lead to better recovery, reduced inflammation and improved gastrointestinal disorders during training (Ferraz et al., 2020; Yang et al., 2022). This is particularly significant for athletes with celiac disease, as polyphenols have been shown to promote overall health and increase athletic performance, and reduce gastrointestinal symptoms while improving gut microbiota (Cannataro et al., 2023). Integrating polyphenol-rich foods or supplements into the diet of celiac athletes could therefore be an effective strategy for enhancing training adaptations.

### 4.4 Other cereals

Sorghum, millet, and teff are gluten-free cereals with important nutritional properties, making them suitable alternatives for celiac patients. Sorghum is characterised by its high fibre content (6.6 g/100 g) and significant amounts of antioxidants, B vitamins, and minerals such as potassium (324 mg/100 g), magnesium (12 mg/100 g), calcium (12 mg/100 g) and zinc (1.6 mg/100 g) (Rinninella et al., 2021). Furthermore, some sorghum varieties are rich in phenolic acids, including ferulic, p-coumaric and vanillic acids, in addition to condensed tannins. These compounds have been demonstrated to contribute to the anti-cancer, cardioprotective and anti-diabetic properties. Millet is another valuable cereal, providing 10 g/100 g of protein, insoluble dietary fibre and 2.6 g/100 g of polyunsaturated fatty acids (PUFAs). It is also a good source of minerals such as magnesium, potassium, selenium as well as folate. In addition, it is rich in both free and bound phenolic acids. Millet's glycaemic index, ranging from 42.7 to 58.3, and it as a beneficial low-GI food, especially for people managing blood sugar levels. Its nutritional value and hypoglycaemic properties highlight its potential in the gluten-free functional food market. Teff, an ancient Ethiopian whole grain, is another gluten-free cereal of interest, with 13.3 g/100 g of protein, 8 g/100 g of fibre, and is particularly rich in iron (7.63 mg/100 g), potassium (427 mg/100 g), zinc (3.63 mg/100 g) and calcium (180 mg/100 g). These cereals present nutritionally dense alternatives to refined gluten-free products and contribute meaningfully to a balanced gluten-free diet (Rinninella et al., 2021).

### 4.5 Pseudocereals

Gluten-free pseudocereals, such as amaranth, quinoa, and buckwheat, represent a valuable dietary strategy for athletes with celiac disease, helping to overcome the nutritional limitations of conventional gluten-free products. These grains are rich in protein, fibre, carbohydrates, and polyunsaturated fatty acids, all of which are fundamental for muscle synthesis and recovery, as well as for maintaining energy metabolism during training. Their high fibre content supports gut health, enhances satiety and contributes to stable blood glucose levels, which are important for endurance performance. Pseudocereals are also a good source of vitamins, including folic acid, riboflavin, vitamin E, and vitamin C. The use of pseudocereals has been shown to address the nutritional deficiencies that are commonly associated with celiac disease and conventional gluten-free products, thereby enhancing their nutritional profile (Rinninella et al., 2021). Despite the potential risks associated with cross-contamination, oats have been shown to offer significant nutritional benefits, including protein, fibre, thiamine, and zinc (Caeiro et al., 2022). Studies have shown that celiac patients who consume oats report similar or even improved symptoms and quality of life compared to those who do not (Cheng and Handu, 2020). Pseudocereals such as quinoa, amaranth and buckwheat are particularly valued for their digestible protein content and high fibre content. Buckwheat has been shown to contain higher levels of fibre than most conventional cereals, while amaranth and quinoa have been identified as sources of complete proteins and a wide range of vitamins and minerals. Furthermore, the bioactive compounds present in these grains, such as polyphenols, saponins, and phytosterols, have anti-inflammatory and antioxidant properties which may mitigate exercise-induced oxidative stress and support intestinal integrity (Caeiro et al., 2022). In this context, the study of Bravi et al. (2024) demonstrated that combining up to 60% quinoa flour and rice flour in gluten-free shortbread biscuits significantly increased the content of dietary fibre, polyphenols, and essential amino acids compared to conventional formulations. This contributes to better gut health, enhanced antioxidant capacity, and a more balanced energy profile. Integrating pseudocereals into a gluten-free diet has been shown to substantially improve nutritional quality while preventing deficiencies in essential micronutrients such as calcium, iron, and zinc. Furthermore, this approach is a valuable nutritional strategy for athletes with celiac disease, enabling them to optimise their protein intake and micronutrient status to support performance and recovery (Rinninella et al., 2021).

## 5 Implication for practice

Athletes with celiac disease face daily challenges in meeting their energy and nutritional needs while maintaining a strict gluten-free diet. This is particularly important for athletes, whose nutritional requirements are high to support optimal performance, muscle recovery, and metabolic balance. However, nutritional deficiencies are often more attributable to inadequate nutrient intake from an imbalanced gluten-free diet than to persistent malabsorption (Mędza and Szlagatys-Sidorkiewicz, 2023). Targeted supplementation with macronutrients, micronutrients and vitamins has been shown to effectively prevent persistent nutritional deficiencies in patients with celiac disease, including athletes (Mikulska et al., 2024). Studies suggest that consuming approximately 2 g of protein and 2 g of fat per kg of body weight, combined with a moderate reduction in carbohydrates, can optimise energy balance, support muscle protein synthesis, and enhance recovery during training (Cannataro et al., 2023). High-quality proteins from pseudocereals, such as quinoa, amaranth and buckwheat, provide a complete amino acid profile and digestible protein, thereby contributing to gut health and glycaemic stability (Rinninella et al., 2021). Micronutrient supplementation has been shown to restore haematological parameters, reduce fatigue and support overall metabolic function. Functional bioactive compounds, such as polyphenols and probiotics, can provide further support by reducing inflammation, modulating oxidative stress and improving intestinal barrier (Pecora et al., 2020; Cannataro et al., 2023). In addition to new dietary approaches, the effective management of celiac disease requires regular clinical follow-up, including psychosocial evaluations (Wieser et al., 2021; Rose et al., 2024). The guidelines of the European Society of Paediatric Gastroenterology, Hepatology and Nutrition (ESPGHAN) also emphasise the importance of individualised follow-up, as early as developmental age, to ensure dietary adherence, prevent complications and ease the transition to adulthood, especially in athletes (Mearin et al., 2022). Finally, new dietary perspectives, such as the use of prebiotics, probiotics, functional foods, and low FODMAP diets, are showing potential in promoting a healthy gut microbiota. Although not yet approved for clinical use, these strategies could in the future be an important support to the gluten-free diet, helping to improve the quality of life of celiac athletes and reduce the risk of complications related to refractory or poorly controlled celiac disease (McDermid et al., 2023). Despite increasing awareness, research on celiac disease in athletes remains limited. Further studies should adopt sport-specific clinical trial designs to assess how gluten-free diets, microbiota modulation, and targeted supplementation after recovery, performance and metabolic efficiency.

## Author contributions

AB: Conceptualization, Writing – original draft. DN: Writing – original draft. PP: Supervision, Writing – original draft, Writing – review & editing. GM: Visualization, Writing – original draft. AP: Supervision, Visualization, Writing – review & editing. Pa.P: Methodology, Visualization, Writing – review & editing. OM: Visualization, Writing – review & editing. AA: Supervision, Visualization, Writing – review & editing.

## Generative AI statement

The authors declare that no Gen AI was utilised in the creation of the manuscript. AI-assisted technologies were employed solely to improve the readability and language of the manuscript. All modifications were carried out under human supervision, with the authors carefully reviewing every change.

## Funding

The authors declare that no financial support was received for the research, authorship, and/or publication of this article.

## Declaration of competing interest

The authors declare that they have no known competing financial interests or personal relationships that could have appeared to influence the work reported in this paper.
